# High-Resolution Diffusion Tensor Spinal Cord MRI Measures as Biomarkers of Disability Progression in a Rodent Model of Progressive Multiple Sclerosis

**DOI:** 10.1371/journal.pone.0160071

**Published:** 2016-07-28

**Authors:** Francesca Gilli, Xi Chen, Andrew R. Pachner, Barjor Gimi

**Affiliations:** 1 Department of Neurology, Geisel School of Medicine at Dartmouth, Hanover, New Hampshire, United States of America; 2 Department of Radiology, Geisel School of Medicine at Dartmouth, Hanover, New Hampshire, United States of America; San Raffaele Scientific Institute, ITALY

## Abstract

Disease in the spinal cord is a major component of disability in multiple sclerosis, yet current techniques of imaging spinal cord injury are insensitive and nonspecific. This study seeks to remove this major impediment to research in multiple sclerosis and other spinal cord diseases by identifying reliable biomarkers of disability progression using diffusion tensor imaging (DTI), a magnetic resonance imaging technique, to evaluate the spinal cord in a model of multiple sclerosis, i.e. the Theiler’s Murine Encephalitis Virus-Induced Demyelinating Disease (TMEV-IDD). Mice with TMEV-IDD with varying levels of clinical disease were imaged using a 9.4T small animal MRI scanner. Axial diffusivity, radial diffusivity, and fractional anisotropy were calculated. Disability was assessed periodically using Rotarod assay and data were expressed as a neurological function index. Correlation was performed between DTI measurements and disability scores. TMEV-IDD mice displayed significant increased neurological deficits over time when compared with controls (*p*<0.0001). Concurrently, the values of fractional anisotropy and axial diffusivity were both decreased compared to controls (both *p*<0.0001), while radial diffusivity was increased (*p*<0.0001). Overall, fractional anisotropy changes were larger in white matter than in grey matter and differences were more pronounced in the ventral region. Lower disability scores were associated with decreased fractional anisotropy values measured in the ventral (r = 0.68; *p*<0.0001) and ventral-lateral (r = 0.70; *p*<0.0001) regions of the white matter. These data demonstrate that DTI measures of the spinal cord contribute to strengthening the association between neuroradiological markers and clinical disability, and support the use of DTI measures in spinal cord imaging in MS patients.

## Introduction

Multiple sclerosis (MS) is a chronic inflammatory disorder of the central nervous system (CNS) and is the most common cause of non-traumatic disability among young adults [1]. MS etiology is currently unknown and its pathogenesis is only partly understood. Multifocal regions of inflammation and myelin destruction accompanied by neuronal degeneration and axonal loss characterized the pathology of MS [2–4]. The disease can remain clinically silent for many years, and permanent neurological disability develops when a threshold of damage is reached and the CNS compensatory resources are exhausted [3]. Much of the disability that develops in MS patients is thought to be due to disease of the spinal cord, which is almost always involved in MS [5].

As symptoms of MS are extremely variable, magnetic resonance imaging (MRI) offers an attractive, noninvasive option for the diagnosis as well as for the longitudinal follow-up of disease progression and/or response to treatments. However, several studies have pointed out that routine MRI of the spinal cord is insensitive and non-specific in monitoring disease progression, as no significant correlation between T2 lesions and MS disability is detected [6, 7]. This lack of reliability of spinal cord imaging in identifying significant disease is a major problem in the clinical management of MS patients. In the present study, we sought to address this gap by testing the hypothesis that diffusion tensor imaging (DTI), an advanced imaging technique, can reliably quantitate MS disease in the spinal cord by using an animal model of progressive MS, i.e. the Theiler’s murine encephalomyelitis virus-induced demyelinating disease (TMEV-IDD).

TMEV-IDD is a demyelinating disease that is induced in susceptible mice by persistent CNS infection with the neurotropic virus, Theiler’s murine encephalomyelitis virus (TMEV), inducing diffuse CNS inflammation and demyelination, mostly in the spinal cord [8, 9]. It is ideally suited as a model of the progressive CNS damage in MS because its pathology and clinical progression are very similar to those observed in humans with MS [10, 11].

DTI provides qualitative and quantitative information about the microarchitecture of white matter (WM). The applications of this technique for characterizing the structural changes that result from lesions in the brain are now well established [12]. More recently, DTI has also been applied to the spinal cord and has been demonstrated to be a similarly valuable tool for assessing the extent of WM damage in different spinal cord-related conditions including MS [13]. Unfortunately, DTI in the spinal cord presents a number of technical difficulties, including respiratory and cardiac motion artefacts and a mismatch in magnetic susceptibility between soft tissue and bone. Advances in MRI such as improved radiofrequency coil design for imaging the spine, better shimming algorithms and rapid imaging methods have contributed to improving the quality of DTI data.

We used DTI to longitudinally extract imaging markers of MS pathology and to correlate these markers with the neurological disability observed in TMEV-IDD mice. DTI data of the spinal cord were acquired with a 9.4 T Agilent small animal imaging system using a small volume coil with extended coverage and homogeneous excitation/detection, an outer volume suppression scheme, and reduced field of view acquisition to provide high in-plane resolution [14, 15].

DTI measures of axial diffusivity, radial diffusivity, and fractional anisotropy were extracted from gray matter as well as from dorsal, ventral, dorsal-lateral and ventral-lateral WM regions. These measures were then correlated with characteristics of TMEV-IDD such as neurological disability and CNS inflammation.

Present data show that improved DTI measures of the spinal cord may contribute to strengthening the association between neuroradiological markers and clinical disability, overcoming the lack of correlation of routine MRI measures with disability, often observed in MS.

## Material and Methods

### Mice

Female SJL/J mice were purchased from the Jackson Laboratory (Bar Harbor, ME). Mice were 4–6 weeks old at initiation of these experiments and were maintained on standard laboratory chow and water *ad libitum*. All animal experiments were approved by the Institutional Animal Care and Use Committee (IACUC) of Dartmouth College.

### Inoculation of mice and humane endpoint

Mice were anesthetized with isofluorane and inoculated in the right cerebral hemisphere with 2x10^5^ plaque-forming units (PFU) of the DA strain of TMEV in a 10 μL volume. Control mice received 10 μL of phosphate-buffered saline (PBS). PFU were determined by a cytopathic effect assay. Blood from each experimental and control mouse at days 24 and 108 post infection (p.i) was collected from the retro-orbital plexus of isofluorane-anesthetized mice; serum was isolated and stored at -20°C. Mice were necropsied at any average of day 190 p.i, or earlier if their severe neurological disability endangered their life.

The humane endpoint in this study, at which the mice were euthanized, was defined as clear signs of severe weakness and inability to get to food or water, quantified as a drop below 10 seconds (s) of Rotarod running time or a loss of 15% bodyweight. Mice were subjected to daily health checks over the entire follow up. In addition, neurological motor functions were scored weekly by the Rotarod test; mice, which dropped from the usual running time of about 100 or more s to 20 s, were monitored twice a week to see if they were showing signs of severe weakness and inability to get to food or water. In order to reduce pain and distress, mice were anesthetized prior to any invasive procedure, including MRI scans. Mice were not treated with analgesics.

Euthanasia was induced by exsanguination/cardiac perfusion, followed by decapitation for necropsy. Briefly, mice were deeply anesthetized with 0.05 to 0.1 mL (depending on the weight of the mouse) of a Ketamine-Xylazine-Acepromazine (20/0.5/0.5 mg/mL) cocktail, injected intraperitoneally. Blood was then obtained by cardiac puncture and perfusions were performed with PBS, to remove the remaining blood from tissues. By the completion of removal of blood and saline perfusion the mouse had expired, and organs of interest were harvested for analyses. Overall, techniques were performed as previously described, including anesthesia, perfusion with PBS, and the collection of blood and other tissues such as spinal cord and brain [16–18].

### Assay of TMEV-specific serum antibody concentrations

Serum anti-viral antibody levels of individual experimental and control mice were measured by enzyme-linked immunosorbent assay (ELISA) with DA antigen [18, 19]. Sera were diluted from 1/500 to 1/4000 in PBS/0.05% Tween/0.5% non-fat dry milk. A horseradish peroxidase-conjugated donkey anti-mouse immunoglobulin G (IgG) (Jackson ImmunoResearch Inc., West Grove, PA, USA) was used as the detecting antibody. A positive control consisting of a pool of high-titer sera from infected mice was used as standard reference sample. The levels of antibody were interpolated from a standard curve constructed by relating the optical densities (OD) produced by dilutions of the standard reference sample, and used on every plate. OD values were converted into arbitrary units (AU) based on the standard curve.

### Real-time RT-PCR for *in situ* IgG production and TMEV load

Expression of IgG and TMEV RNA was ascertained as previously described with specific primers and probes for murine IgG1 and TMEV [17, 20]. Total RNA was isolated from fresh, homogenized tissue samples using TRIzol^®^ (Invitrogen, Carlsbad, CA, USA). Reverse transcription (RT) was performed using the qScript cDNA SuperMix (Quanta Biosciences, Gaithersburg, MD, USA) following the manufacturer’s instructions. cDNA was then used as a template for real time polymerase chain reaction (PCR) analysis based on the 5’ nuclease assay. Mouse glyceraldehyde phosphate dehydrogenase (GAPDH) was used as a reference gene. TaqMan^®^ real time PCR assays (Lifetechnologies, Grand Island, NY, USA) were used as primers and probes for GAPDH, whereas custom primers and probes were used for amplification of IgG1 and TMEV.

Relative mRNA expression level of IgG1 was analyzed by using the 2^-ΔCt^ method, in which ΔCt is (Ct_target_—Ct_GAPDH_). In the determination of viral load in tissue, a standard curve was utilized and the threshold cycle (Ct) values of the samples were plotted against known TMEV concentrations.

### Rotarod testing for progressive disability

Progressive disability in mice was assessed with the Rotarod test (Columbus Instruments, Columbus, OH, USA), as previously described [16]. The Rotarod assay is the neurobehavioral assay of choice for the TMEV-IDD model of MS in many laboratories,[21–25] and it was chosen because proven to be more sensitive than standard clinical assessment methods, e.g. neurological score evaluation and bodyweight loss, for measuring progressive neurological dysfunctions.[26]

Rotarod data were expressed as a neurological function index (NFI). The NFI value at any time point was the mean of the last 3 time indices divided by the mean time indices from day 15 to day 45 p.i. Time indices were the time on the Rotarod for that day divided by the mean of the 2 maximum times for that mouse.

Standard clinical assessments like weight loss and clinical grading of TMEV-IDD [9] were also regularly performed (S1 Fig).

### Mice selection for imaging

Twenty-nine mice were selected for imaging from a wider group of 60 TMEV-infected mice. Selections for the first group, the longitudinal group, were made randomly, while selections for the second group were made on the basis of measured disability at the final time point. The first set of twelve TMEV-infected mice, randomly selected at day 30 p.i, was longitudinally followed from day 90 to day 190 p.i with repeat spinal cord DTI every 30 days (a total of 4 imaging time-points for each mouse). Six sham-infected controls were included as well. These mice were called the “longitudinal group”.

A second set of 20 TMEV-infected mice, called “the day 190 group”, was selected and scanned at day 190 p.i. Those mice were chosen because they had the highest (n = 10) or lowest (n = 10) disability indices. Three mice overlapped the first set of mice, bringing the total number of infected mice tested in the present study to 29.

### DTI-MRI

MRI was performed on a 9.4 T Agilent small animal imaging system equipped with a 30 mm transceiver coil (www.agilent.com). Mice were anesthetized using an isoflurane–oxygen mixture of 2.5% for induction and 1.5–2.0% for maintenance. Temperature and respiration was monitored throughout the scan.

**Fig 1:**
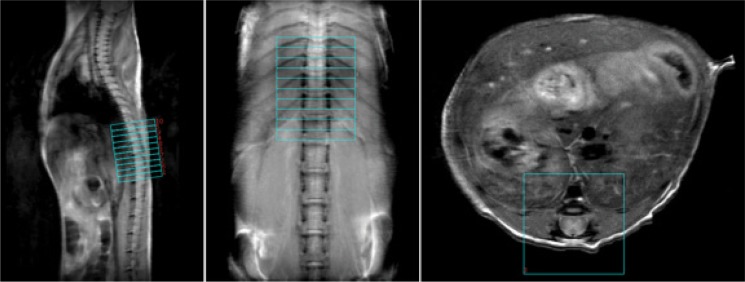
Anatomical spinal cord images of a representative control mouse. DTI slices were marked using the 8th-13th ribs as landmark. On the axial plane a reduced field-of-view (FOV) for the DTI acquisition was shown, with outer-volume signals suppressed.

First, single sagittal and coronal slices were acquired using a multiple spin echo sequence using the following parameters: repetition time TR = 1000 ms, echo time TE = 10 ms, number of echoes = 8, number of averages = 4, slice thickness = 4 mm, in-plane resolution = 117 μm x 117 μm. These two views were chosen to give a landmark of 8th-13th ribs for localization of desired vertebra segments (Fig 1).

Next, using landmarks from the anatomical images, axial images covering vertebra T10—L1 (Fig 1) were acquired using the multiple spin-echo sequence with the following parameters: repetition time TR = 1600 ms, echo time TE = 8 ms, number of echoes = 8, number of averages = 8, ten slices 1 mm thick with in-plane resolution of 100 μm x 100 μm.

DTI images were obtained with respiratory gating, using a spin echo sequence with two b0 averages and 6 gradient directions with b = 1000 s/mm^2^, effective TR = 1600–2400 ms, TE = 18 ms, number of slices = 10 (interleaved), slice thickness = 1 mm, and in-plane resolution = 80 μm x 80 μm. Outer volume suppression (OVS) was achieved using a high-order hyperbolic secant adiabatic pulse to saturate a slab on the left and the right of the field-of-view (each 10 mm thick) and another slab over the lung region.

To minimize motion artefacts, the mouse was secured in the animal holder in a supine position with the head secured in a nose cone. The respiration period was maintained in a range of 0.8–1.2 s and respiratory gating was applied for the DTI study. Acquisition was triggered with a 100 ms delay and 5 slices, with acquisition duration of 1.92 ms (96 data points with spectral width = 50 kHz) and single-shot repetition time of 100 ms, were acquired within 500 ms during the exhalation period. A total of 10 slices with thickness = 1 mm were acquired interleaved in two respiration periods with an effective TR = 1.6–2.4 s.

### Data and statistical analyses

**Fig 2:**
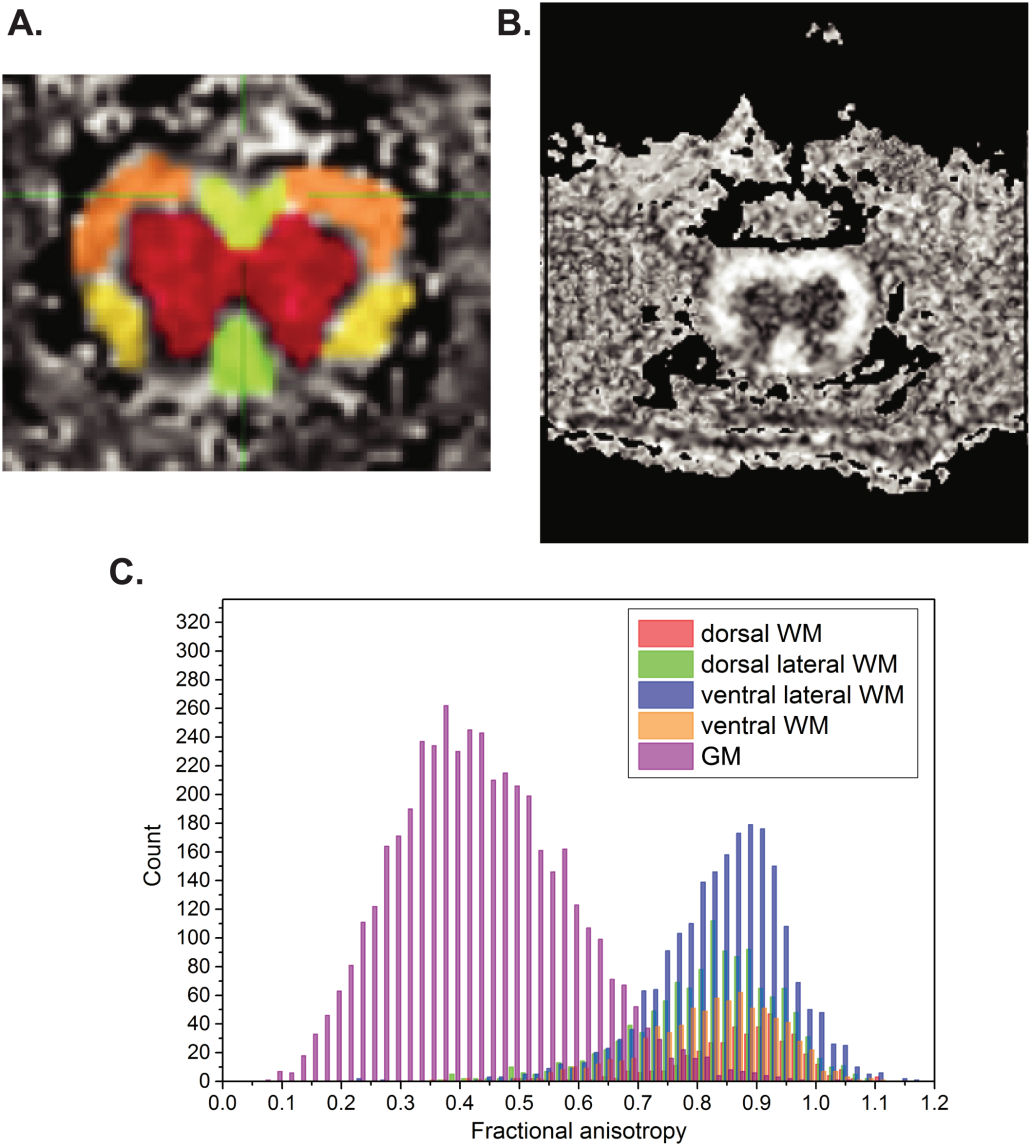
Defining regions of interest (ROI) on transverse DTI images. A) DTI measures were obtained for different ROIs in the spinal cord, namely dorsal, dorsal-lateral areas (both in yellow areas), ventral and ventral-lateral (both in orange areas) white matter and gray matter. B and C) White matter and grey matter were segmented based on contrast, and WM sub-regions were manually outlined on the FA maps

DTI data were processed using Diffusion Toolkit in TrackVis software. Eigenvalues and eigenvectors were extracted from the DTI data to calculate axial diffusivity (AD), radial diffusivity (RD), and fractional anisotropy (FA). DTI is based on differential patterns of water molecule diffusion occurring along the length of the axon. Water molecule movement that is perpendicular to the length of the axon (RD) is compared to movement that is parallel (AD). The asymmetry in molecule movements is termed FA. Overall, the most frequently used diffusivity parameter is FA, which is a good index of fiber integrity. AD is generally regarded as a marker of axonal integrity, and RD is considered a marker of myelination. All these DTI measures were obtained for several regions-of-interest (ROIs) in the spinal cord, namely dorsal, dorsal-lateral, ventral and ventral-lateral white matter (WM) and gray matter (GM) (Fig 2A). ROIs were manually defined in slices corresponding to spinal cord levels L2-L5. These ROIs were analyzed independently and were defined using the AFNI software.[27] WM and GM were separated based on contrast (Fig 2B and 2C), and WM sub-regions were manually outlined on the FA maps, as previously described by Underwood and colleagues. [28]

During MRI scans mice were carefully monitored by the same operator in order to make sure there was no physiological instability, e.g. movement of mice that could be observed by the respiratory curve, and the respiration rate was kept stable at 50–70 /minute. The FA maps were reconstructed immediately after the scans on the scanning console to check for image quality such as blurring.

Physiological instability was detected in 1 to 3 mice per time point. Those mice (n = 18) were rescanned after 3 days. There is no data exclusion in the group analysis after all scans were done

All statistical analyses were performed using either SPSS software (IBM, Armonk, NY, USA) or GraphPad PRISM version 6.00 (GraphPad Software, San Diego, CA, USA). Either a one-way or two-way analysis of variance (ANOVA) followed by Dunn’s or Tukey HSD post-doc test respectively, was used for multiple comparisons. Group-to-group differences were assessed using Mann-Whitney U test. Spearman rank correlation was used to measure the degree of association between DTI metrics, neurological function (NFI), and biological parameters. All reported *p* values are based on two-tailed statistical tests, with a significance level of 0.05.

## Results

### Analysis of the longitudinal group

This group consisted of 12 TMEV-infected mice and 6 sham-infected controls, randomly selected at day 30 p.i, that underwent repeat spinal cord DTI every 30 days (a total of 4 imaging time-points for each mouse) from day 90 to day 190 p.i.

#### Clinical and Biological Data in the longitudinal group

**Fig 3:**
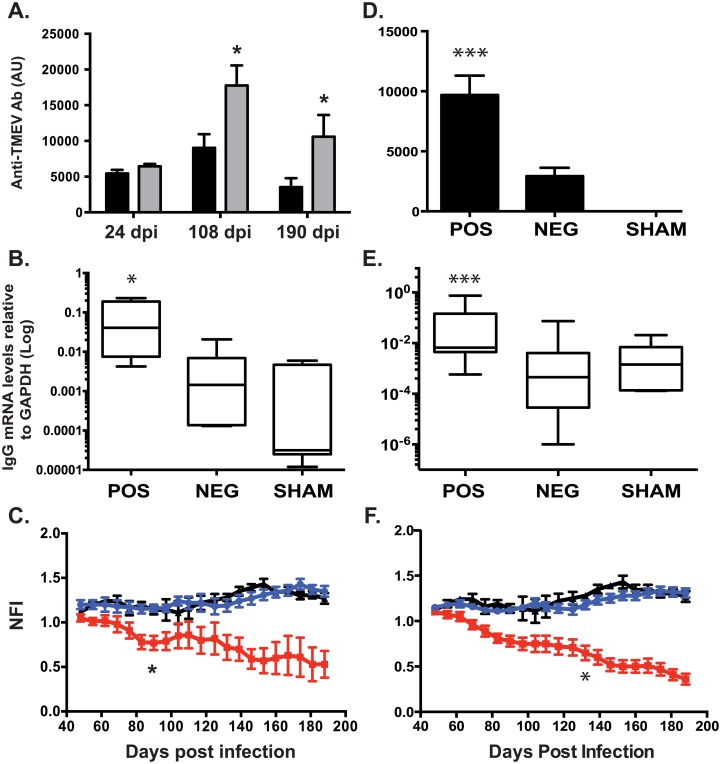
Longitudinal and cross-sectional studies of biological and clinical data in TMEV infected mice. Levels of anti-TMEV antibodies in blood (A, D), IgG mRNA expression in spinal cord (B, E) and disability indexes (C, F) were evaluated in n = 18 mice (n = 12 infected with TMEV and 6 sham controls) followed longitudinally over a period of 190 days post infection (p.i) (A-C), as well as in n = 35 mice (n = 29 infected with TMEV and 6 sham controls) evaluated at day 190 p.i (D-F). At the end of the longitudinal follow-up, the presence of TMEV RNA was detected in the spinal cord of 4/12 infected mice (TMEV PCR-positive mice). (A) Serum anti-TMEV antibody levels were measured by ELISA at three different time points, i.e. day 24, 108 and 190 p.i. Levels of serum anti-TMEV antibody were higher in TMEV PCR-positive (grey columns) compared with TMEV PCR-negative (black columns) mice on both day 108 and 190 p.i, whereas no difference was detected on day 24 p.i. (B) *In situ* production of IgG was measured by real time RT-PCR for IgG mRNA in the spinal cord. Levels of IgG mRNA were higher in TMEV PCR-positive mice compared with both TMEV PCR-negative and sham mice. (C) TMEV PCR-positive mice (red squares) displayed significantly increased neurological deficits over time when compared with both TMEV PCR-negative (blue circles) and sham-treated (black triangles) mice. The difference was statistically significant starting at day 83 p.i up to the end of the follow-up. There was no difference comparing TMEV PCR-negative and sham-treated mice. Within the group of 29 TMEV infected mice analyzed in the cross-sectional study, 16 were TMEV PCR-negative and 13 were TMEV PCR-positive. (D) Serum anti-TMEV antibody levels were significantly higher in TMEV PCR-positive mice compared with both TMEV PCR-negative and control mice. Likewise, (E) levels of IgG mRNA in the spinal cord were higher in TMEV PCR-positive mice compared with both TMEV PCR-negative and sham mice. Moreover, (F) TMEV PCR-positive mice (red squares) displayed significantly increased neurological deficits over time when compared with both TMEV PCR-negative (blue circles) and sham-treated (black triangles) mice. The difference was statistically significant starting at day 130 p.i up to the end of the follow-up. Data are shown either as mean value ± SEM (A and D) or as Log Min to Max value (B and E). P values are shown as follows: * P<0.05 ** P < 0.001 *** P<0.0001.

Within the group of 18 mice (12 infected with TMEV and 6 sham-infected) followed longitudinally, all 12 infected mice were positive for serum anti-TMEV antibodies on day 24 p.i indicating that a TMEV infection had actually occurred. However, at the end of the 190-day follow-up, 8 of the 12 infected mice had cleared the virus, as PCR for TMEV RNA in spinal cords from these mice was negative. Accordingly, levels of serum anti-TMEV antibody were significantly lower in PCR negative compared with PCR positive mice on both day 108 and 190 p.i (both *p*≤0.04) (Fig 3A and Table 1). PCR for TMEV RNA and anti-TMEV antibodies by ELISA were negative in the sham control group.

**Table 1:** Biological parameters in n = 18 mice longitudinally followed with spinal cord DTI. Values are mean ± standard error of the mean (SEM).

			Spinal Cord IgG mRNA	TMEV Ab Serum			Rotarod Assay (190 dpi)		
Group	# mice	Spinal Cord Viral load (copy number in 0.5 μg RNA)	Sham-Normalized Relative Expression	Day 24	Day 108	Day 190	NFI	Sham Normalized NFI	Running time (sec)
TMEV-PCR Negative	8	2.0±1.0	7.79±4.63	5.44x10^3^ ±5.14x10^2^	9.02x10^3^ ±1.93x10^3^	3.53x10^3^ ±1.25x10^3^	1.28±0.05	0.97±0.04	2.13x10^2^ ±8.81
TMEV-PCR Positive	4	2.67x10^6^ ±8.10x10^5^	3.83x10^2^ ±2.53x10^2^	6.46x10^3^ ±3.28x10^2^	17.77x10^3^ ±2.80x10^3^	10.59x10^3^ ±3.04x10^3^	0.53±0.15	0.40±0.11	1.02x10^2^ ±28.11
Sham	6	0±0	/	0±0	0±0	0±0	1.27±0.05	/	2.04x10^2^ ±15.97

*In situ* production of IgG was measured by real time RT-PCR for IgG mRNA in the spinal cord. Spinal cords were collected at necropsy at an average of 190 days p.i. Levels of IgG expression in the spinal cord were significantly higher in TMEV PCR-positive mice compared with both TMEV PCR-negative and sham mice (both *p*≤0.038) (Fig 3B and Table 1).

TMEV PCR-positive mice displayed significantly increased neurological deficits over time, as assessed with the Rotarod assay, when compared with both TMEV PCR-negative and sham-treated mice (Fig 3C); ANOVA analysis revealed a significant effect of persistent TMEV infection (F_(2, 315)_ = 179.9, *p*<0.0001). The following Tukey HSD post-hoc multiple comparison test revealed that TMEV PCR-positive mice had significantly lower NFI than those for TMEV PCR-negative and sham mice (*p*≤0.01). The difference was statistically significant starting at day 83 p.i up to the end of the follow-up. There was no difference comparing TMEV PCR-negative and sham-treated mice (Fig 3C).

#### DTI data in the longitudinal group

The values of FA, AD, and RD in TMEV PCR-positive animals showed changes in different ROIs: FA and AD were both significantly decreased compared to TMEV PCR-negative and sham controls, while RD was increased. Overall, FA changes in TMEV PCR-positive mice were larger in WM than in GM.

**Fig 4:**
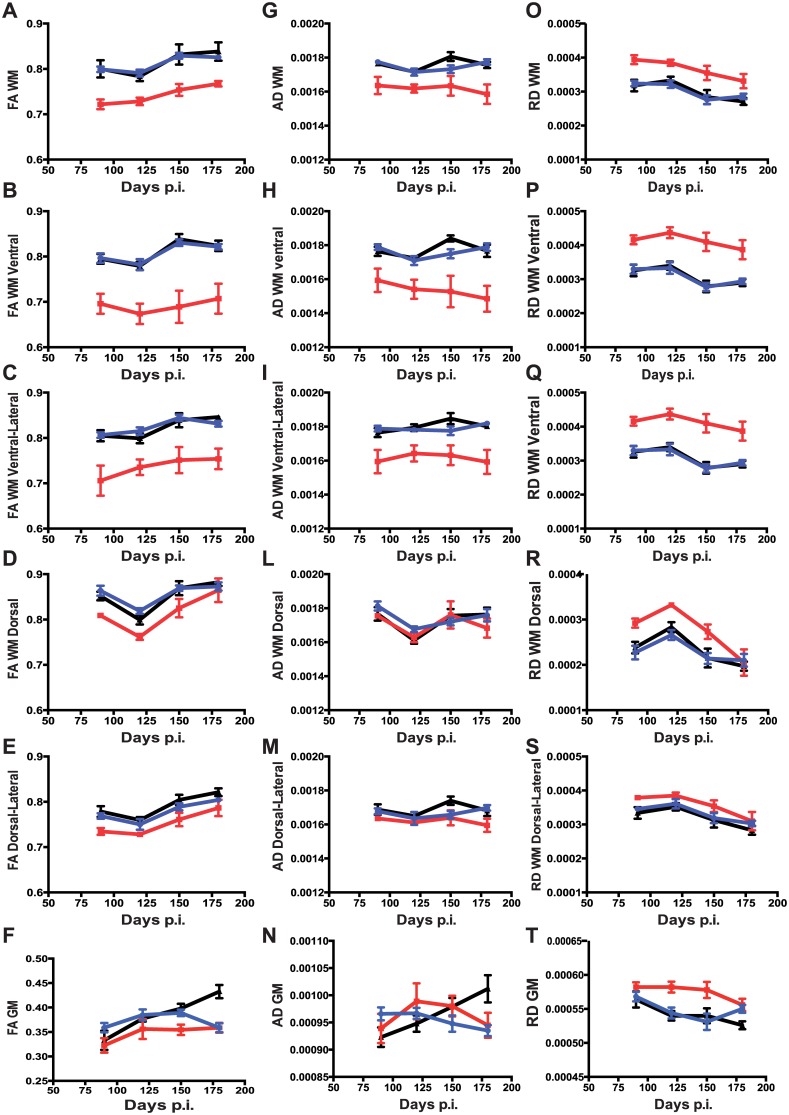
Longitudinal study of DTI measures in TMEV infected mice. Group-based trajectory models of fractional anisotropy FA (A-F), axial diffusivity AD (G-N) and radial diffusivity RD (M-T) measures in different white matter regions-of-interest (ROI) and total grey matter. DTI measures were acquired from n = 18 mice (n = 4 TMEV PCR-positive, n = 8 TMEV PCR-negative and 6 sham mice). Mice were longitudinally followed from day 90 to day 190 p.i with repeat spinal cord DTI every 30 days, i.e. a total of 4 imaging time-points for each mouse. Solid red circles indicate TMEV PCR-positive mice, solid blue circles indicate TMEV PCR-negative mice, and solid black triangles indicate sham controls. Data are shown as mean value at each time point ± SEM.

Considering total WM, two-way ANOVA yielded a main effect for persistent TMEV infection, F_(2, 59)_ = 46.50, *p*<0.0001, such that the average FA values were significantly lower in TMEV PCR-positive compared to TMEV PCR-negative and sham mice (Tukey HSD test, all *p*<0.0001). The main effect of time p.i was significant as well (F_(3, 59)_ = 12.57, *p*<0.0001), but no interaction effect was observed (F_(6, 59)_ = 0.3954, *p* = 0.8778), indicating that the effect of persistent TMEV infection on FA values was not significantly different across the analyzed time points (Fig 4A and S2 Fig.).

Differences between the TMEV PCR-positive, TMEV PCR-negative and sham animals were more pronounced in the ventral region of the WM. FA values were significantly lower in the TMEV PCR-positive group at every time point (Tukey HSD test, all *p*<0.01) (Fig 4B). The ventral-lateral FA values in the WM were similar to those obtained in the ventral region (Fig 4C). The reduced FA in the ventral and ventral-lateral regions of the WM was observed to be due to changes in both AD and RD because both parameters were significantly altered in TMEV PCR-positive mice compared with both TMEV PCR-negative and sham animals (Fig 4H, 4P, 4I and 4Q).

In the dorsal and dorsal-lateral regions of the WM, differences were significantly smaller and not always statistically significant (Fig 4D, 4E, 4L, 4M, 4R and 4S).

In the total GM, the pattern of all subgroups was closely similar, but in sham-treated mice we observed an increase in FA throughout the follow-up, leading to a significant difference at the last time point, i.e. 190 days p.i (Fig 4F). At day 190 p.i, FA values were significantly lower in TMEV PCR-positive compared with sham mice (*p*<0.0001). FA values in TMEV PCR-positive mice were not different from TMEV PCR-negative mice (*p* = 0.134).

### Analysis of the day 190 group

The total number of mice used for statistical analysis were n = 35, i.e. 29 TMEV-infected and 6 sham-treated mice.

#### Clinical and Biological Data in the day 190 group

Within the group of 29 TMEV-infected mice, 16 were negative by PCR for TMEV RNA in their spinal cords, whereas the remaining 13 mice had positive spinal cord TMEV RNA readings. The mean viral load in the spinal cord of the latter group was 1,027,472 TMEV copy numbers (SEM = 418,037; range 1,906–4,368,698) (Table 2).

**Table 2:** Biological parameters in n = 35 mice tested with spinal cord DTI at day 190 post infection. Values are mean ± standard error of the mean (SEM).

			Spinal Cord IgG mRNA	TMEV Ab Serum			Rotarod Assay (190 dpi)		
Group	# mice	Spinal Cord Viral load (copy number in 0.5 μg RNA)	Sham-Normalized Relative Expression	Day 24	Day 108	Day 190	NFI	Sham Normalized NFI	Running time (sec)
TMEV-PCR Negative	16	5.0±4.0	28.76 ± 22.17	6.11x10^3^ ±5.91x10^2^	8.94x10^3^ ±1.47x10^3^	2.93x10^3^ ±7.10x10^2^	1.31±0.05	0.99±0.04	2.15x10^2^ ±5.92
TMEV-PCR Positive	13	1.03x10^3^ ±4.18x10^3^	5.03x10^2^ ±2.38x10^2^	7.24x10^3^ ±9.13x10^2^	19.02x10^3^ ±2.39x10^3^	9.69x10^3^ ±1.62x10^3^	0.36±0.06	0.27±0.04	52.9±12.72
Sham	6	0±0	/	0±0	0±0	0±0	1.27±0.05	/	2.04x10^2^±15.97

Levels of serum anti-TMEV antibody were significantly higher in TMEV PCR-positive compared with TMEV PCR-negative mice (*p* = 0.0002) (Fig 3D). Similarly, levels of IgG expression in the spinal cord were significantly higher in TMEV PCR-positive mice compared with both TMEV PCR-negative and sham mice (*p =* 0.0006) (Fig 3E and Table 2). There was no statistical difference between TMEV PCR-negative and sham-treated mice (*p* = 0.268).

Once again, TMEV PCR-positive mice displayed increased neurological deficits when compared with both TMEV PCR-negative and sham-treated mice, with a significant difference found between TMEV PCR-positive mice and both TMEV PCR-negative and sham animals (F_(2, 40)_ = 67.97, *p*<0.0001) (Fig 3F). Post-hoc multiple comparison test confirmed that TMEV PCR-positive mice had significantly lower NFI than both TMEV PCR-negative and sham mice (*p*≤0.01). In addition, the NFI scores at day 190 p.i were significantly lower for TMEV PCR-positive mice compared with those negative or sham (both *p*<0.0001). TMEV PCR-positive mice had an average NFI score of 0.36 ± 0.06 compared to 1.31 ± 0.05 and 1.27 ± 0.05 in TMEV PCR-negative and sham mice, respectively (Table 2).

Low NFI scores were confirmed by clinical symptoms consistent with chronic progressive paralysis, including gait abnormalities, spastic paralysis, and tail and hindlimb weakness, quantified in S1 Fig. using a TMEV-IDD clinical scoring system [9].

#### DTI data in the day 190 group

**Fig 5:**
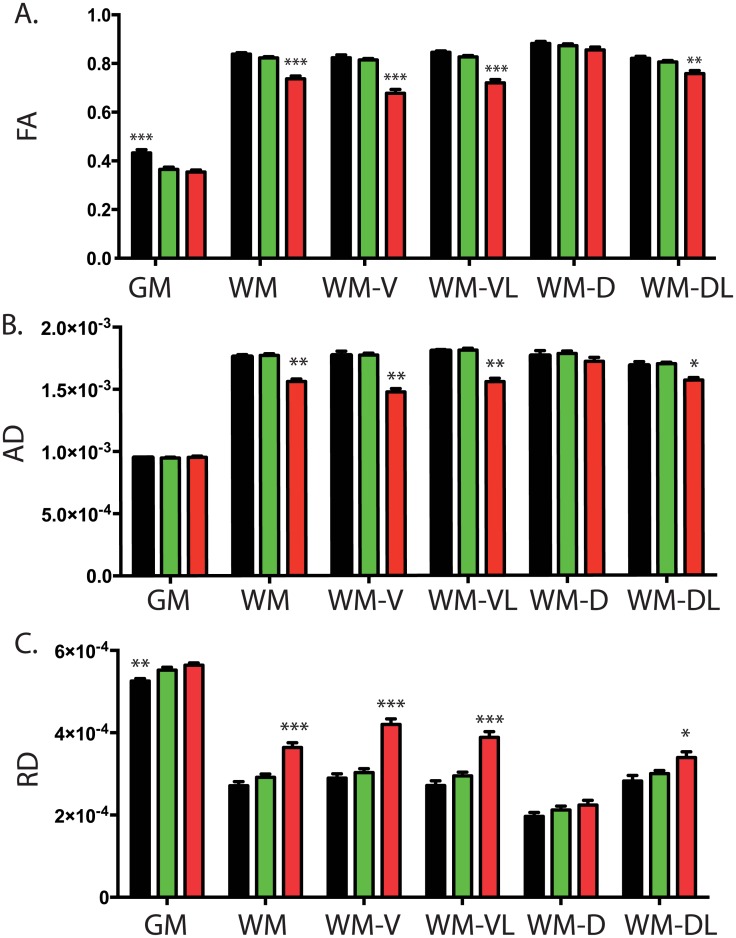
Cross-sectional study of DTI measures in late TMEV infected mice. DTI measures of spinal cord were analyzed in n = 35 mice (n = 16 TMEV PCR-negative, 13 TMEV PCR-positive and 6 sham controls). Measures were acquired at day 190 p.i. (A) In comparison with controls (black bars), mice with TMEV-IDD had lower FA values in the grey matter (GM), regardless of their TMEV PCR-positive (red bars) or negative (green bars) status. In contrast, FA values measured in the white matter (WM) were lower in TMEV PCR-positive (red bars) mice compared with both control mice (black bars) and TMEV PCR-negative mice (green bars). Overall data show significant differences among WM regions: damage was primarily observed in the ventral (WM-V) and ventral lateral (WM-VL) tracts, whereas no damage was observed in the WM dorsal (WM-D) and dorsal later (WM-DL) tracts. Both AD (B) and RD (C) measures showed changes in TMEV PCR-positive mice, with a diffuse pattern of involvement in the WM, primarily in the ventral (WM-V) and ventral later (WM-VL) regions. Data are shown as mean value + SEM. P values are shown as follows: * P<0.05 ** P < 0.001 *** P<0.0001.

DTI parameters of spinal cord were acquired and analyzed at day 190 p.i. In comparison with sham controls, mice with TMEV-IDD had lower FA values in the GM (ANOVA *p =* 0.0015), regardless of their TMEV PCR-positive or negative status (both *p*≤0.0005) (Fig 5A). There was no difference between TMEV PCR-positive and negative animals (*p* = 0.494).

In contrast, FA values measured in the WM were significantly lower in spinal cords of TMEV PCR-positive mice (ANOVA *p* = 0.0001), i.e. mice with significant disability, compared with both sham-treated mice and TMEV PCR-negative mice (both *p*≤0.0001) (Fig 5A). No difference was observed comparing FA values in sham controls and TMEV PCR-negative mice, i.e. mice with no evident disability (*p* = 0.071).

The ANOVA demonstrated overall significant differences between WM regions for FA in TMEV PCR-positive mice (*p*<0.0001). Particularly, data showed primarily damage in the axial plane, being ventral and ventral lateral (both *p*<0.0001). No FA differences were observed in the WM dorsal tract (*p* = 0.353), while a slight difference was reported in the dorsal lateral tract of the WM (*p* = 0.004) (Fig 5A).

Fig 5B and 5C show that both AD and RD terms showed changes in TMEV PCR-positive mice (both *p*<0.0001), with a diffuse pattern of involvement in the WM (both *p*<0.0001). Differences between the TMEV PCR-positive, TMEV PCR-negative and sham animals were more pronounced in the ventral and ventral lateral regions of the WM (all *p*<0.0001). In the dorsal and dorsal-lateral regions of the WM, differences were significantly smaller and not always statistically significant (all *p*≥0.022).

In the GM, the observed changes in FA were mainly due to changes in RD (*p* = 0.008) rather than AD (*p* = 0.058) (Fig 5B and 5C). Notably, RD values were significantly higher in both TMEV PCR-positive and negative mice compared with sham controls (both *p*≤0.031). RD values in TMEV PCR-positive mice were not different from TMEV PCR-negative mice (*p* = 0.091).

### Correlational of DTI and biological measures in all mice undergoing MRI analysis

We found a weak-to-moderate correlation between FA and viral load in the spinal cord in a number of the ROIs. In particular, higher viral loads were associated with decreased FA values in the GM (r = -0-41; *p* = 0.014) as well as in the WM (r = -0.70; *p*<0.0001), excluding the dorsal region (r = -0.14; *p* = 0.425). Similarly, a larger expression of IgG mRNA in the spinal cord correlated with decreased FA values in the ventral (r = -0.46; *p* = 0.005) and ventral-lateral (r = -0.40; *p* = 0.017) regions of the WM, but not in the dorsal (r = 0.073; *p* = 0.67) and dorsal-lateral (r = 0.065; *p* = 0.71) regions as well as in the GM (r = 0.004; *p* = 0.98) (S3 Fig).

**Fig 6:**
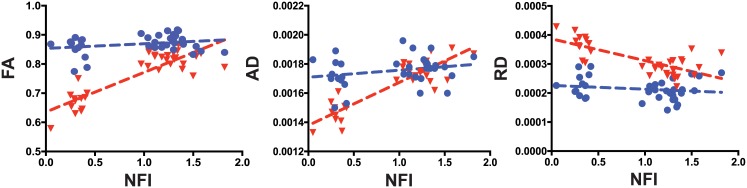
Correlation between DTI measures in the white matter and disability scores. Blue circles indicate DTI measures in the white matter dorsal region (WM-D), and red triangles indicate DTI measures in the white matter ventral region (WM-V). DTI measures, i.e. (A) FA, (B) AD, and (C) RD exhibited a significant relationship with disability scores (neurological function index -NFI) in the white matter ventral (WM-V) region, but not in the white matter dorsal (WM-D) region. The blue dotted line shows regression line of “WM-D plots” and the red dotted line shows “WM-V plots”.

In addition, lower NFI scores were associated with decreased FA values measured in the ventral (r = 0.68; *p*<0.0001) and ventral-lateral (r = 0.70; *p*<0.0001) regions of the WM, but not in the dorsal region of the WM (r = 0.08; *p* = 0.625) and the GM (r = 0.24; *p* = 0.170). There was only a slight correlation (r = 0.41; *p* = 0.013) between NFI and FA values in the dorsal-lateral region of the WM (Fig 6).

Increased levels of serum anti-TMEV antibodies correlated with decreased FA values measured in the ventral and ventral-lateral regions of the WM. Correlation was significant at day 108 and 190 p.i, whereas no statistically significant correlation was observed at day 24 p.i. A weak correlation was also observed in the dorsal-lateral region of the WM and in the GM. There was no correlation in the dorsal region of the WM (S4 Fig).

Viral loads, *in situ* IgG production, NFI and serum anti-TMEV antibodies significantly correlated with AD values in all ROIs of the WM, except the dorsal one. The same parameters also correlated with RD values in the GM and all ROIs of the WM except the dorsal and dorsal-lateral regions.

## Discussion

The potential of DTI to detect spinal cord abnormalities in humans has already been demonstrated [29–34]. FA measure had the strongest evidence of utility, with moderate quality evidence for its use as a biomarker showing correlation with disability in several clinical pathologies, including MS (for review see [35]). Likewise RD has been identified as a predictive factor of outcome in a cohort of MS patients with cervical cord relapse [32]. So far, the evidence is insufficient to determine if spinal cord DTI can be used as a diagnostic test, biomarker, or prognostic marker in a clinical context [35]. However, although only small cohorts of patients have been analyzed and different sequences and methodologies have been used, the data and current experience suggest that DTI has a potential to develop as a useful and feasible technique in early detection of disability progression in patients.

Promising DTI data have also been collected in rodents. Authors reported the first *in vivo* DTI study in the spinal cord of experimental allergic encephalomyelitis (EAE) mice, observing significantly decreased AD compared to the control group [36]. Further studies have been published on the EAE model, analyzing disease course, correlations with histology [37, 38], clinical score [39], and treatment efficacy [40].

Although TMEV-IDD clearly represents one of the best models for MS disease progression, currently there are no published studies providing data on *in vivo* DTI in the spinal cord of TMEV-IDD mice, a gap that needs to be filled in order to confirm the utility of the spinal cord DTI in clinical setting. On these grounds, the present study aimed to investigate the biomarker potential of DTI measures for the assessment of disease progression in MS through spinal cord analyses in TMEV-IDD. Towards this goal, DTI measures were examined in both longitudinal and cross-sectional DTI datasets generated from persistently TMEV-infected mice, transiently TMEV-infected mice and sham controls.

In this study, mice persistently infected with TMEV, defined as a positive PCR result for TMEV mRNA in the spinal cord at necropsy, developed progressive neurological disability, as measured by the Rotarod test, beginning in the third month post-infection. DTI measures in these mice showed significant changes in different ROIs: FA and AD were both decreased compared with control mice, while RD was increased. Among the three DTI measures, we chose to focus particularly on FA, as the correlation of FA with neurological impairment appears to be strongest in human neurodegenerative conditions. Moreover, FA is a function of AD and RD and it represents a summary measure of microstructural integrity. Thus, FA can be potentially used as a biomarker of global CNS damage in TMEV-IDD.

Overall, in the current longitudinal study FA changes were larger in the WM than in the GM, with no significant differences across the analyzed time points. In persistently infected mice relative to sham mice, lower FA values were observed already at the earlier time point, i.e. day 90 p.i, remaining decreased throughout the follow up. Also, FA differences between infected and control mice were more pronounced in the ventral regions of the spinal cord, where all DTI metrics, i.e. FA, AD and RD, were significantly altered in persistently infected mice compared with controls. Interestingly, in previous pathology studies on TMEV-IDD, the localization of T cells and macrophages infiltrates was shown to be restricted primarily to the ventral spinal cord region [41]. Likewise, studies have shown that in TMEV-IDD, demyelination is principally confined to the ventral and ventral-lateral WM on one or both sides of the ventral medial fissure [42]. Thus, our present DTI data fully confirm previous pathology studies, which have accordingly demonstrated primarily injury in the axial plane being ventral and ventral-lateral [41–43].

Another interesting result of the study was that FA values in the WM increased with time in both the sham and the persistently TMEV infected mice. Axonal swelling was suggested as a possible interpretation of this observation. However, this seems an unlikely explanation, since increasing FA values were observed in both TMEV infected mice and sham controls. Thus, this phenomenon is unlikely to be associated with any TMEV-IDD pathological process. On the other hand, the increase of FA values over time may reflect a dynamic process in the spinal cord over the first 7–10 months of the mouse’s life. Recent studies of mice 1 to 10 months of age demonstrated that the spinal cord significantly increases in mass over time as a result of a late CNS maturation process [22, 44]. The increase of mass was shown to be disproportionately rapid compared to that of the actual cell number, which resulted in reduced cellular densities for both neuron and non-neuron, including glia [44]. This change may be the consequence of an increase in mean neuronal size, axonal extension, and especially myelination [44]. These observations fully support our present DTI data, showing a constant increase over time of FA values and a concurrent decrease of RD values, possibly reflecting the myelination process. Similar data have been reported in humans too, where it has been shown that during childhood and adolescence, WM FA increases over time in different CNS regions, including the spinal cord [45, 46].

We next focused on cross-sectional data as a mean to obtain an evaluation of the relationship between DTI measures and biological parameters, as well as between DTI measures and neurological deficit in TMEV-IDD. Hence, DTI measures of spinal cord integrity were acquired and analyzed in a larger group of persistently TMEV-infected mice at day 190 p.i, i.e. at a late, neurodegenerative stage of the disease. These mice had markedly impaired neurologic function compared with sham controls, as well as high level of local and peripheral antibody response to the virus. Moreover, in agreement with the longitudinal data, cross-sectional results showed primarily damage in the ventral and ventral-lateral regions of the WM, where lower FA values were measured in persistently infected mice.

We correlated DTI measures with different biological parameters such as viral load, *in situ* IgG expression and levels of anti-TMEV antibodies in blood. We found a weak-to-moderate correlation between FA and viral load in the spinal cord. In particular, higher viral loads were associated with decreased FA values in the ventral regions of the WM. In this same region we also found that decreased FA values correlated with a higher expression of *in situ* IgG expression. These findings confirm, once again, that the ventral regions of the WM are the primary sites of neuronal damage due to multifactorial TMEV-induced injury. Moreover, the increased *in situ* IgG response to the virus appears to be a primary cause of the TMEV-mediated CNS damage. This agrees well with our previous studies showing that disease progression highly correlates with intrathecal antibody production [16], and that many plasma cells, i.e. cells that secrete large volumes of antibodies, accumulate and reside in the CNS parenchyma of persistently TMEV-infected mice [42].

We have also shown that increased levels of serum anti-TMEV antibodies correlated with decreased FA values measured in the ventral regions of the WM. However, significant correlations with later disability were found only for anti-TMEV antibody levels measured during an advanced chronic phase of TMEV-IDD, and not when antibodies were measured during a preclinical stage of the disease. Notably, moderately elevated levels of serum anti-TMEV antibodies were detected in all TMEV-infected mice at day 24 p.i, meaning that mice had been actually exposed to the virus. In contrast, high levels of anti-TMEV antibodies were detected at later time points only in mice with a persistent TMEV infection of the CNS.

Anti-TMEV antibodies are more likely not an independent variable but depend on viral persistence; since progressive disease is seen only in persistently infected mice, anti-TMEV antibodies are perforce increased in more disabled mice. Thus, the relationship between CNS damage and peripheral antibodies in TMEV-IDD is not clear, and further studies are needed to confirm the existence of an actual link between the peripheral anti-TMEV immune response and disease progression.

A final interesting result of the study is that DTI measures significantly correlated with late disability scores, suggesting a potential clinical value of this approach. The three DTI measures, i.e. FA, AD, and RD, measured in the ventral regions of the WM, showed significant correlation with disability scores, and especially FA was found to be sensitive to disease progression. A clear weakness in our study is the lack of histologic samples to confirm the relationship between DTI measures and neurological disability in TMEV-IDD. However, previous studies from our laboratory and others have found significant correlations between histology variables and Rotarod scores.[21, 47–50]. Thus, it is reasonable to expect that a significant relationship would be also observed between the histological and DTI results.

Neurological disability in TMEV-IDD was previously shown to correlate with spinal cord atrophy [22]. Currently little is known about the relationships and differences between macrostructural spinal cord atrophy and microstructural diffusion changes in contributing to functional impairment in mice. However, DTI measures in the spinal cord seem to provide a more precocious characterization of mice with disability. Indeed, spinal cord atrophy was detected late in the follow up, i.e. 6 to 12 months p.i [22], while DTI measures were here shown to be altered already by 3 months p.i. Instead, functional disability was apparent by 3–4 months p.i, with continued worsening over time. Unfortunately, our current study design does not allow proper evaluation of FA as a predictor of future disability progression in mice with subclinical involvement since our earlier DTI scan was acquired in mice when the disease was already clinically evident. Thus, evaluation of FA in other studies will be needed to provide further clarity to its potential as a predictive biomarker of disability progression.

## Conclusions

The present study shows that in TMEV-IDD the most abnormal DTI measures are present in the ventral regions of the spinal cord WM. The association between the DTI indices and the levels of *in situ* IgG, serum anti-TMEV antibody and viral load suggests the involvement of these molecules in promoting microstructural changes in the CNS. Finally, the association between disability and the ventral WM FA indices suggests that DTI might be a useful indicator of neurological disability. Results from these studies might provide a basis for the early detection of changes related to a wide range of neurodegenerative disorders and the identification of periods in the lifespan when interventions will potentially have their greatest impact.

## Supporting Information

S1 Fig Body weights and average clinical scores over time. (A) The sham treated (n = 6, black circles) and TMEV PCR-negative (n = 16, blue triangles) mice showed an increase in body weight throughout the study. The TMEV PCR-positive mice (n = 13, red squares) displayed a decrease in body weights; by day 160 post-infection (pi), body weights had decreased significantly compared with those of TMEV PCR negative and sham mice. (B) Clinical scores were assessed weekly beginning on day -8 pi and ending on day +188 pi. Based on the TMEV-IDD clinical score system, TMEV PCR-positive mice (red squares) showed an increase in impairment severity throughout the course of the study; by day 117 pi, clinical scores were significantly higher than the average of both the TMEV PCR-negative mice (blue triangles) and sham controls (black circles). No differences in TMEV-IDD clinical scores were noted in TMEV PCR-negative and sham-treated mice. Data are mean ± SEM; *p<0.05, **p<0.001, compared to sham controls.
(EPS)

S2 Fig Time course of DTI and T2 weighted signals in the spinal cord of a representative mouse with severe progressive disability. A) Axial scans were obtained from a mouse in the TMEV PCR-positive group, 90, 120, 150 and 190 days post TMEV infection (dpi). The images include quantitative maps from a DTI experiment, as well as the T2-weighted “reference” image from DTI. This representative mouse shows a significant decrease of FA (B), as well as a severe disability progression over the entire follow up (C).
(EPS)

S3 Fig Correlation between fractional anisotropy and biological parameters in the grey and white matter. Blue squares indicate measures in the total grey matter (GM), and red triangles indicate measures in the total white matter (WM). (A and B) Viral loads in the spinal cord exhibited a significant relationship with FA in both the GM and the WM (both p<0.014). (C and D) A significant correlation was also showed between *in situ* IgG mRNA expression and FA in the total WM (p = 0.04), but not in the GM (*p* = 0.98).
(EPS)

S4 Fig Correlation analysis of DTI measures and peripheral anti-TMEV antibody levels. Correlation models of fractional anisotropy FA (A-F), axial diffusivity AD (G-N) and radial diffusivity RD (M-T) measures in different white matter regions-of-interest (ROI) and total grey matter. DTI measures were acquired from n = 18 mice (n = 4 TMEV PCR-positive, n = 8 TMEV PCR-negative and 6 sham mice). Mice were eye bled and tested for the presence of anti-TMEV antibodies at day 24, 108, and 190 p.i. Overall, FA, AD, and RD changes showed a significant correlation (p<0.03) with anti-TMEV antibody levels in the grey matter (F, N, T) and in all ROIs of the white matter (A, B, C, E, G, H, I, M, O, P, Q, S), with the exception of the dorsal region (D, L, R). Correlation was significant at day 108 (red squares) and 190 (blue triangles) p.i, whereas no statistically significant correlation was observed at day 24 (black circles) p.i.
(EPS)

## Abbreviations

DTI: diffusion tensor imaging
TMEV: Theiler’s murine encephalomyelitis virus
TMEV-IDD: Theiler’s Murine Encephalitis Virus-Induced Demyelinating Disease
MS: multiple sclerosis
CNS: central nervous system
MRI: magnetic resonance imaging
WM: white matter
GM: grey matter
PFU: plaque-forming units
p.i: post infection
PBS: phosphate-buffered saline
ELISA: enzyme-linked immunosorbent assay
IgG: immunoglobulin G
OD: optical densities
AU: arbitrary units
RNA: ribonucleic acid
cDNA: copy deoxyribonucleic acid
RT: reverse transcription
GAPDH: glyceraldehyde phosphate dehydrogenase
Ct: threshold cycle
NFI: neurological function index
AD: axial diffusivity
RD: radial diffusivity
FA: fractional anisotropy
ROIs: regions-of-interest
ANOVA: analysis of variance
s: seconds
mg: milligrams
mL: milliliter
μL: microliter
